# 3D Bio-Printability of Hybrid Pre-Crosslinked Hydrogels

**DOI:** 10.3390/ijms222413481

**Published:** 2021-12-15

**Authors:** Cartwright Nelson, Slesha Tuladhar, Loren Launen, Ahasan Habib

**Affiliations:** 1 Department of Sustainable Product Design and Architecture, Keene State College, Keene, NH 03435, USA; cartwright.nelson@keene.edu (C.N.); slesha.tuladhar@keene.edu (S.T.); 2 Department of Biology, Keene State College, Keene, NH 03435, USA; llaunen@keene.edu

**Keywords:** 3D bioprinting, shape fidelity, hybrid hydrogel, pre-crosslinking, rheology, CMC

## Abstract

Maintaining shape fidelity of 3D bio-printed scaffolds with soft biomaterials is an ongoing challenge. Here, a rheological investigation focusing on identifying useful physical and mechanical properties directly related to the geometric fidelity of 3D bio-printed scaffolds is presented. To ensure during- and post-printing shape fidelity of the scaffolds, various percentages of Carboxymethyl Cellulose (CMC) (viscosity enhancer) and different calcium salts (CaCl_2_ and CaSO_4_, physical cross-linkers) were mixed into alginate before extrusion to realize shape fidelity. The overall solid content of Alginate-Carboxymethyl Cellulose (CMC) was limited to 6%. A set of rheological tests, e.g., flow curves, amplitude tests, and three interval thixotropic tests, were performed to identify and compare the shear-thinning capacity, gelation points, and recovery rate of various compositions. The geometrical fidelity of the fabricated scaffolds was defined by printability and collapse tests. The effect of using multiple cross-linkers simultaneously was assessed. Various large-scale scaffolds were fabricated (up to 5.0 cm) using a pre-crosslinked hybrid. Scaffolds were assessed for the ability to support the growth of Escherichia coli using the Most Probable Number technique to quantify bacteria immediately after inoculation and 24 h later. This pre-crosslinking-based rheological property controlling technique can open a new avenue for 3D bio-fabrication of scaffolds, ensuring proper geometry.

## 1. Introduction

In recent years, the horizon of tissue engineering and regenerative medicine (TERM) has been expanding vastly due to a revolutionary fabrication technology called 3D bioprinting and the availability of biomaterials compatible with this technique [1]. Various 3D bioprinting processes such as extrusion-based [2,3,4], inkjet [5,6], and laser-assisted [7,8] bioprinting processes provide spatial control and repeatability of material deposition for attaining design specific 3D tissue scaffolds. The extrusion-based bioprinting process has gained comparatively more popularity because of its capability to release a diverse range of materials and allow higher cell density [9]. Naturally growing polymeric materials and hydrogels are used frequently to fabricate highly porous 3D constructs that serve as temporary structural support for growing isolated cells [10]. Hydrogels are considered mainstream scaffolding materials to prepare bio-inks due to their high water content, ability to closely mimic the extra cellular matrix, flexible structure, desirable and controllable biocompatibility, and biodegradability [11,12,13].

Achieving controlled spatiality of the fabricated 3D scaffold with hydrogel materials is still challenging [14]. Different hydrogel materials are blended to prepare a hybrid hydrogel with an expectation of achieving the required properties of bio-ink and final structure [15,16]. Those properties can be categorized as rheological, mechanical, shape fidelity, interlayer attraction, cytotoxicity, and degradability [17]. Rheological properties are critical during and after the hydrogel extrusion through a small nozzle orifice. Material with improper rheological properties may not develop enough mechanical properties, such as yield strength, to maintain dimensional accuracy [18] after getting released from the nozzle. Researchers are controlling the yield strength of hydrogel materials using viscosity modifiers [19,20,21], changing temperature [22,23,24], using an external cross-linker [25,26], incorporating sacrificial materials [27], and controlling intrinsic rheological properties of the hydrogels [28,29]. It becomes a conflicting characteristic when a hydrogel needs to create a comfortable habitat for encapsulated cells during extrusion, and it needs to have proper rheological properties to maintain the post-printing shape fidelity [30,31]. Sometimes, increasing the percentage of the solid content of hydrogel materials may not guarantee geometric fidelity [29,32]. Moreover, this higher percentage of solid content may build a roadblock for necessary gas and nutrient flows [33,34]. Using sacrificial support materials poses the challenge of sufficient removal during the incubation periods [35]. Therefore, we hypothesized that applying pre-crosslinker to a hybrid hydrogel having a lesser amount of solid content (≤6% *w*/*v*) can result in equivalent or improved mechanical and rheological properties, and eventually better printability and geometric fidelity, relative to previous designs.

Various bi- and trivalent cations such as Ca^2+^ [26,36], Ba^2+^ [37], Zn^2+^ [37], Sr^2+^ [38], Ga^3+^ [39], Al^3+^ [40] have been used as physical crosslinkers. Among them, CaCl_2_ and CaSO_4_ are the most common [41]. Sometimes, physical cross-linkers can be applied while formulating the hydrogel materials before 3D bioprinting. This approach is defined as the pre-crosslinking of hydrogels [42]. In previous work [42], alginate was pre-crosslinked with CaCl_2_ and CaSO_4,_ respectively. Having similar rheological properties, pre-crosslinked hydrogels showed lower shape fidelity. In another work, 2% alginate was pre-crosslinked with various percentages of CaCO_3_ and a build height up to 5.0 mm was reported [35]. Various amounts of alginate along with graphene oxide was pre-crosslinked with CaCl_2_ having an intention to define some measurable rheological parameters and corresponding shape fidelity of the fabricated scaffold [43]. Alginate and alginate-hydroxy apatite (HA) were pre-crosslinked with 1% CaSO_4,_ and the pre-osteoblast cell was encapsulated to compare with chitosan and chitosan-HA [44]. The results showed that Chitosan-HA has better bone-forming capability than alginate-HA. However, the analysis of printability and shape fidelity was not compared and reported there. 

Although CaCl_2_ and CaSO_4_ are both physical cross-linkers and show a linear viscosity upon rheological tests, they are quite different when it comes to printability and holding their structure. If we were to look at a molecular level, CaCl_2_ is readily soluble in water which often leads to an uncontrolled release of Ca_2+_ ions and therefore results in a heterogeneous cross-linking. This leads to the use of higher extrusion pressure while printing, which can be fatal to the cells. In contrast, sulfate salts have lower solubility and exhibit uniform cross-linking over a period. This makes CaSO_4_ readily printable at a lower extrusion pressure and easier to handle than CaCl_2_ [41,42]. Similarly, since CaSO_4_ has a higher Young’s modulus and equilibrium modulus, it has better mechanical properties. As a result, compositions cross-linked with CaSO_4_ exhibit better structural integrity after printing than those cross-linked with CaCl_2_.

**Figure 1:**
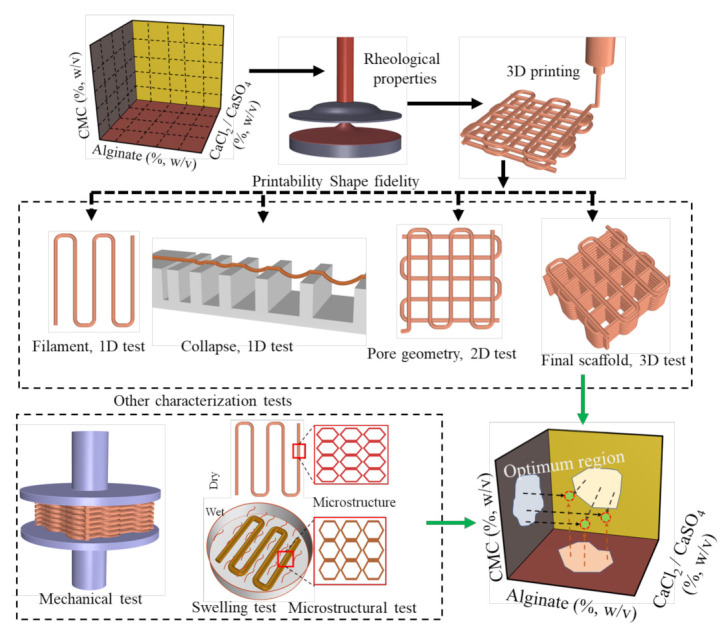
Schematic representation of the research: Finding out the optimum material compositions.

Alginate is the most common linear polysaccharide, broadly used to formulate bio-ink for the extrusion-based 3D bioprinting process because of its controllable rheological properties, biocompatibility, biodegradability, hydrophilicity, micro-porosity, and physical cross-linking [41,45]. Carboxymethyl cellulose (CMC) is a cellulose derivative and a polysaccharide. It is soluble in water, has a high molecular weight, and is used to modify viscosity [46]. Moreover, the binding of CMC’s matrix protein assists in cell migration and cell attachment [47]. Several researchers have combined CMC with alginate to obtain better physical and mechanical properties [46]. To our best knowledge, for the first time, we proposed hybrid hydrogels consisting of alginate and carboxymethyl cellulose (CMC) (high and low viscosity) for the extrusion-based bioprinting process [48,49,50,51]. To improve the rheological properties and achieve a clinically relevant build height (≥10 mm) of the printed scaffold, ensuring better shape fidelity of hybrid hydrogels, we used a pre-crosslinking approach. As base hydrogel materials, we used alginate (high and low viscosity) and carboxymethyl cellulose (CMC) with ≤6% solid content, and CaCl_2_ and CaSO_4_ as pre-crosslinkers. Ca^2+^ ion is known to help in maintenance, motility, transportation, and also cell differentiation processes in not only eukaryotic cells, but also prokaryotes. One of the downsides to being bio-compatible, however, is the possibility of being able to grow contaminating bacteria [52]. A set of characterization tests such as rheological, mechanical, swelling, and microstructural tests were conducted to determine the effect of the viscosity modifier and pre-crosslinker. Then, the effect of rheological and mechanical behaviors of the scaffolds fabricated with pre-crosslinked hydrogels were assessed with respect to the printability and the shape fidelity. Pre-crosslinked hybrid hydrogels were used to fabricate large scaffolds ensuring geometric fidelity. One candidate material was tested for the ability to support the growth of Escherichia coli bacteria. An overview of this research aimed at developing a novel bio-ink is shown in Figure 1. 

## 2. Materials and Methods

### 2.1. Hybrid Hydrogel Preparation

Low (viscosity = 15–25 cps of 1% in water) and medium (viscosity ≥2000 cps of 2% in water) viscous Alginate (A) (alginic acid sodium salt from brown algae) and carboxymethyl cellulose (CMC) (pH: 6.80) (Sigma-Aldrich, St. Louis, MO, USA) were used as biomaterials to prepare the bio-ink. Alginate, a negatively charged linear copolymer, is a common biopolymer, composed of (1-4)-linked β-Dmannuronic (M) and α-Lguluronic acids (G). It is soluble in water and exhibits high biocompatibility. The G-block of this material supports forming gels, and GM and M blocks increase flexibility. Here we used low and medium viscous (LV, MV) alginate simultaneously to ensure both a cell-friendly environment and a high enough cross-linking rate to control shape fidelity [53]. Carboxymethylcellulose (CMC, C) is another naturally or chemically derived anionic water-soluble biopolymer. CMC is a copolymer of β-D-glucose and β-D-glucopyranose-2-O-(carboxymethyl)-mono-sodium salt, which is connected via β-1,4-glucosidic bonds [54]. This material is non-toxic and non-allergenic in nature and is widely used as a thickener [55]. Each glucose monomer has three hydroxyl groups which can be substituted by a carboxyl group. More substitution of the hydroxyl group by carboxyl makes the cellulose more soluble, thick, and stable [54]. Pre- and post-crosslinking were done using calcium chloride (CaCl2, CL) and calcium sulphate (CaSO4, CS) (Sigma-Aldrich, St. Louis, MO, USA). The formulated hybrid hydrogels are shown in Table 1, where the number represents weight percentages of the comprising components. Food colors were used to differentiate the compositions.

### 2.2. Rheological Properties Analysis

Rheological properties were analyzed using a rotational rheometer (MCR 102, Anton Paar, Graz, Austria) with a parallel plate geometry (25.0 mm flat plate). All measurements were recorded with a 1.0 mm plate–plate gap width at room temperature (25 °C). Hydrogels were prepared as explained in Section 2.1 and placed on the Peltier module. Rheological measurements were performed at room temperature in anticipation of using room temperature during our extrusion process. It facilitates the quick gelation of the deposited filament [30]. The type of rheological tests conducted and related process variables are listed in Table 2.

Using the following Power–Law Equation, compositions’ flow characteristic parameters can be determined [56].

(1) $$\eta =K\dot{\gamma }n{-}^{1}$$

where, η is the viscosity, $\dot{\gamma }$ is the shear rate, and *K* and *n* are the shear-thinning coefficients. The linear region of the shear rate vs. viscosity plot will be used to interpolate *n*, and the intersection of the curve at zero-shear rate will identify K. The value of *n* determines the shape of the curve and, more importantly, defines the shear-thinning (if *n* < 1) behavior of the candidate materials. 

The shear rate experienced by the hydrogel at the nozzle wall during the extrusion can be estimated using the following equation [57,58]:

(2) $$\dot{\gamma }\mathrm{NN}=\left(3+\frac{1}{n}\right)\frac{\bar{\nu }}{R}$$

where $\bar{\nu }$ is the average extrusion velocity, which is often considered as the print speed or nozzle moving speed. Ideally, the filament thickness can be controlled by the synchronized extrusion of material [58]; however, in most bioprinting processes, the filament diameter and the material volume eventually vary even if the pressure and the parameters influencing the extrusion materials are kept constant [59]. In this paper, we found a similar phenomenon, i.e., extrusion velocity was always larger than the print head speed, eventually producing larger filament width than the needle diameter. The ratio of this enlargement is defined as the velocity correction factor, which is used to determine the $\bar{v}$. Once the shear rate is estimated using the estimated velocity, the corresponding shear stress can be determined using the following equation:

(3) $$\tau =\eta \dot{\gamma }=K{\dot{\gamma }}^{n}$$

where τ is the shear stress measured on the bio-ink. The spatial distribution of the extrusion velocity profile across the nozzle cross-section can be estimated using the following equation:

(4) $$v=\frac{n}{n+1}{\left(\frac{\Delta p}{2LK}\right)}^{\frac{1}{n}}\left(R^{\frac{n+1}{n}}-r^{\frac{n+1}{n}}\right)$$

where Δp is the applied pressure, L is the nozzle length, and r is the distance of any location from the nozzle center. The corresponding shear rate can be predicted using the estimated extrusion velocity using Equation (2). The distribution of viscosity and shear stress can be estimated using the predicted shear rate using Equations (1) and (3), respectively.

### 2.3. Mechanical Test

The compressive moduli of the fabricated scaffolds (dimensions, *n* = 3) were determined by uniaxial compression force with a universal testing machine (UTM, ADMET, eXpert 2610, Norwood, MA, USA) at room temperature. A compression force was applied at a displacement rate of 0.25 mm per second until 3000 N was reached. 

### 2.4. Swelling Test

The swelling rate is defined as the percentage increase in the weight of the hydrogel due to water absorption. As a set of filaments was extruded, and before storing them in liquid media, all the filament weights were recorded and denoted as dry weight (*Wd*). Filaments were immersed into 4% (*w*/*v*) CaCl_2_ for 15 min. Then, filaments were immersed in water. The weights of the filaments were recorded after 15, 30, and 45 min to identify the changes of geometric fidelity right after the incubation. The wet weight is denoted *Ww*. Using the following equation, % of swelling rate of these filaments is determined:

(5) $$\mathrm{Swelling}\;\mathrm{rate}=\frac{W_{w}-W_{d}}{W_{d}}\times 100\%$$

### 2.5. 3D Printing and Printability Analysis

**Figure 2:**
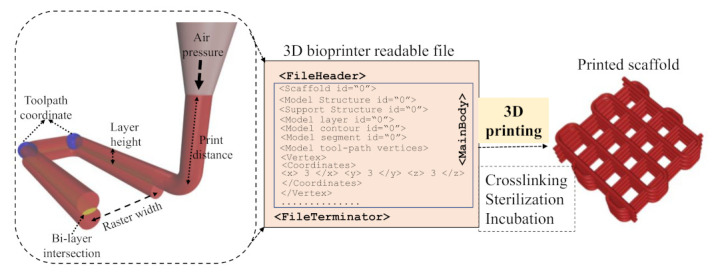
Schematic representation of 3D bioprinting process.

A BioX (CELLINK, Boston, MA, USA) three-axis 3D bioprinter was used to extrude hydrogel and fabricate scaffolds. After preparing hybrid hydrogels (Section 2.1), they were loaded into 3.0 mL disposable syringes and extruded pneumatically on a stationary build plane. Various printing parameters shown in Table 3 can control the deposition rate of the material. Rhino 6.0 (https://www.rhino3d.com, accessed on 10 October 2021), a Computer-Aided Design (CAD) software, was used to design and define the vectorized toolpath of a scaffold. Slicer (https://www.slicer.org, accessed on, 10 October 2021 ), a G-code generator software, was used to generate a Bio-X compatible file including the toolpath coordinates and all process parameters to construct the scaffold. We used a layer-upon-layer method to deposit materials. The partial physical cross-link of the fabricated scaffold after the print was confirmed by a spray of 4% (*w*/*v*) CaCl2. The overall scaffold fabrication process is schematically shown in Figure 2.

#### 2.5.1. Filament Morphology and Printability

The extruded filament should demonstrate a smooth surface and constant width, which will subsequently create regular grids and square holes. However, hydrogel with a dominating liquid-like state may fuse the deposited filament, create circular holes for bi-layer geometry, and eventually close the pore. The circularity of an enclosed area is defined using the following Equation:

(6) $$C=\frac{4\pi A_{a}}{L^{2}}$$

where *L* and $A_{a}$ are the perimeter and the actual area of the enclosed area, respectively. The circularity is 1 for a circle, while it is $\frac{\pi }{4}\;$for a square shape measure from Equation (5). Therefore, the printability (Pr) [21] of the hydrogel is defined using the following Equation:

(7) $$P_{r}=\frac{\pi }{4}\frac{1}{C}=\frac{L^{2}}{16A_{a}}$$

**Figure 3:**
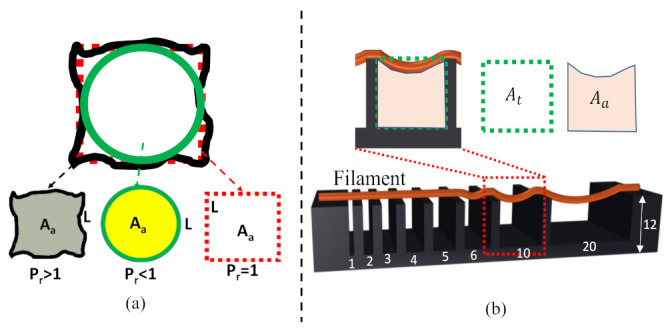
(**a**) Representation of printability, and (**b**) Theory of Collapse test.

The under gelation (Pr < 1), ideal gelation (Pr = 1), and over gelation (Pr > 1) can be defined using the Pr value, as shown in Figure 3a. To determine the printability, various scaffolds having 1.0, 2.5-, and 4.0-mm raster widths (filament to filament distance) were fabricated with a zig-zag pattern using all compositions. The objective of determining printability is to identify the shape-holding capacity of the deposited material. Pore and filament images were captured using the CK Olympus bright field microscope (Tokyo, Japan) and analyzed using ImageJ software. 

#### 2.5.2. Collapse Test

A platform having a variable pillar-to-pillar distance from 1 to 20 mm (1, 2, 3, 4, 5, 6, 10, 20 mm) was designed and manufactured using our in-house 3D printer to perform the collapse test, as shown in Figure 3b. Identifying the collapse rate can indicate the shape-holding capacity of the deposited filament and the deflection rate of a suspended filament. The prepared hydrogel compositions were deposited, and images were captured using the CK Olympus bright field microscope (Tokyo, Japan). They were then analyzed using ImageJ software. The area after the deflection and un-deflected area for collapse tests are shown in Figure 3b. The collapse rate was determined using the following Equation:

(8) $$Rate=\frac{A_{a}}{A_{t}}\times 100\%$$

### 2.6. Scanning Electronics Microscope

The microstructures of thin films generated by various compositions were analyzed by scanning electron microscopy (Tescan Lyra 3 GMU, Brno, Czech Republic). Samples were focused on the surface and cross-sections of the films. The accelerating voltage, spot size (SS), and working distance (WD) used in this imaging are 3 kV, 5.73 × 10^−9^ and 3.6 mm, respectively. All samples were coated with 15 nm Au/Pd using an Anatech Hummer V sputter coater (Sparks, NV, USA). Samples were mounted on ½ inch diameter pin-type stubs and mounting cubes.

### 2.7. Bacterial Growth Analysis

The ability of the composition A_3_C_3_CS_0.5_CL_0.5_ to support 24 h growth of *Escherichia coli* (a common bacterial model organism) was determined using a 3-tube Most Probable Numbers assay [60]. A total of 6 samples consisting of 0.5 g of scaffold in 4.5 mL of sterile water were inoculated with 25 microliters of a 10^−1^ dilution of an overnight *E. coli* culture (5 mL of Tryptic Soy Broth, 37 °C, 240 rpm). Of these, 3 samples were immediately subjected to MPN analysis (time zero), and 3 samples were incubated at 37 °C for 24 h and then subjected to MPN analysis. MPN analysis was conducted as follows. 

At each time point, each replicate sample was used to prepare a 10-fold serial dilution in 4.5 mL of sterile water, yielding 3 dilution series of 10^−1^ to 10^−10^. A 0.5 mL sample of each dilution tube was then used to inoculate 3 tubes containing 4.5 mL of Tryptic Soy Broth. These tubes were grown overnight, shaking at 240 rpm, 37 °C. Turbidity was scored as positive for growth, clear liquid as negative for growth. The MPN pattern was then evaluated, and the MPN was determined using the FDA MPN calculator [61]. 

### 2.8. Statistical Analysis

A format of “mean ± standard deviation” was used to represent data. The evaluation of the statistical significance of the difference of various factors is conducted at a significance level of *p* = 0.05 with a two-way ANOVA. All calculations were done with *n* = 3 unless otherwise stated. Statistical software Minitab 18.0 (State College, PA, USA) and Origin Pro 2021b (Northampton, MA, USA) were used to perform quantitative and graphical analysis. For the bacterial growth assay, the mean MPN and standard error were determined for each time point, and the means were tested using a paired T-test to determine whether they were the same or different using a critical *p*-value of 5%.

## 3. Result

### 3.1. Rheological Test

The rheological properties, i.e., viscosities and shear stress of compositions with pre-crosslinked alginate and CMC, were assessed by different rheological measurements. The impact of the different concentrations of cross-linkers and CMC on rheological behavior with respect to the shear rate was determined by the following tests.

#### 3.1.1. Flow Curve/Shear Thinning Behavior

**Figure 4:**
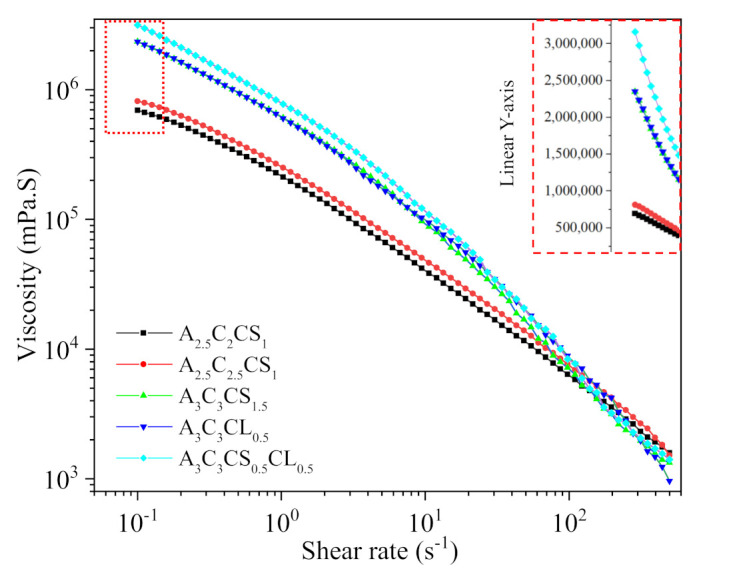
Viscosities of various compositions with respect to the shear rate. Each data point was calculated averaging three sample points.

The shear-thinning coefficients such as *n* and *K*, as shown in Table 4, were determined from the linear region of graphs shown in Figure 4 using Equation (1). For all compositions, we found *n* < 1, which implies all have shear thinning behavior. The actual filament width indicated the solid content and cross-linker dependency. Filaments fabricated with compositions $A_{3}C_{3}{\mathrm{CS}}_{0.5}{\mathrm{CL}}_{0.5}$, $A_{3}C_{3}{\mathrm{CS}}_{1.5}$, and $A_{3}C_{3}{\mathrm{CL}}_{0.5}\;$showed almost similar deviation (64%,67%, and 68%, respectively) from the nozzle diameter. $A_{2.5}C_{2.5}{\mathrm{CS}}_{1}$ and $A_{2.5}C_{2}{\mathrm{CS}}_{1}$ showed 84% and 141% difference with respect to the nozzle diameter. Therefore, $A_{3}C_{3}{\mathrm{CS}}_{0.5}{\mathrm{CL}}_{0.5}$, $A_{3}C_{3}{\mathrm{CS}}_{1.5}$, and $A_{3}C_{3}{\mathrm{CL}}_{0.5}$ showed better shape holding capacity than compositions formed with less solid content and CaSO_4_. The velocity correction factor and the corresponding average velocity were calculated based on this change in the filament width with respect to the nozzle diameter. The values of *n*, K, and average velocity were used to estimate the shear rate in the nozzle during extrusion using Equation (2). The viscosity and shear stresses were calculated using Equations (1) and (3), respectively. The results are shown in Table 1. 

**Figure 5:**
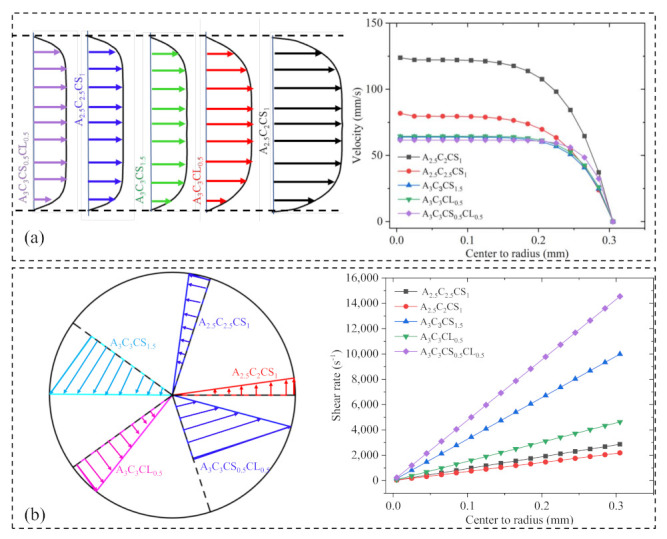
(**a**) Velocity and (**b**) shear stress distribution of various compositions. Velocity and shear rate distribution were calculated based on the shear-thinning factors *n*, K, and filament diameters changed due to the applied pressure. (**a**) represents the width of velocity distribution is viscosity dependent, and (**b**) represents high viscous composition will have more shear stress near the nozzle wall.

The value of K increases with an increase in solid content and cross-linker percentage. The shear rate value signifies the rate at which the material passes through the nozzle, which correlates to the shear stress experienced by the material. From the flow curve, it is clear that with increasing shear rate, the viscosity for $A_{3}C_{3}{\mathrm{CS}}_{1.5}$, $A_{3}C_{3}{\mathrm{CL}}_{0.5},\;{\mathrm{and}\;A}_{3}C_{3}{\mathrm{CS}}_{0.5}{\mathrm{CL}}_{0.5}$ reduced with respect to $A_{2.5}C_{2.5}{\mathrm{CS}}_{1}$ and $A_{2.5}C_{2}{\mathrm{CS}}_{1}$. The shear rate value at the extrusion and corresponding shear stress and velocity supported this behavior. Composition-wise velocity distributions and corresponding shear rate and viscosity distributions are shown in Figure 5.

#### 3.1.2. Storage and Loss Modulus: Gelation Point

**Figure 6:**
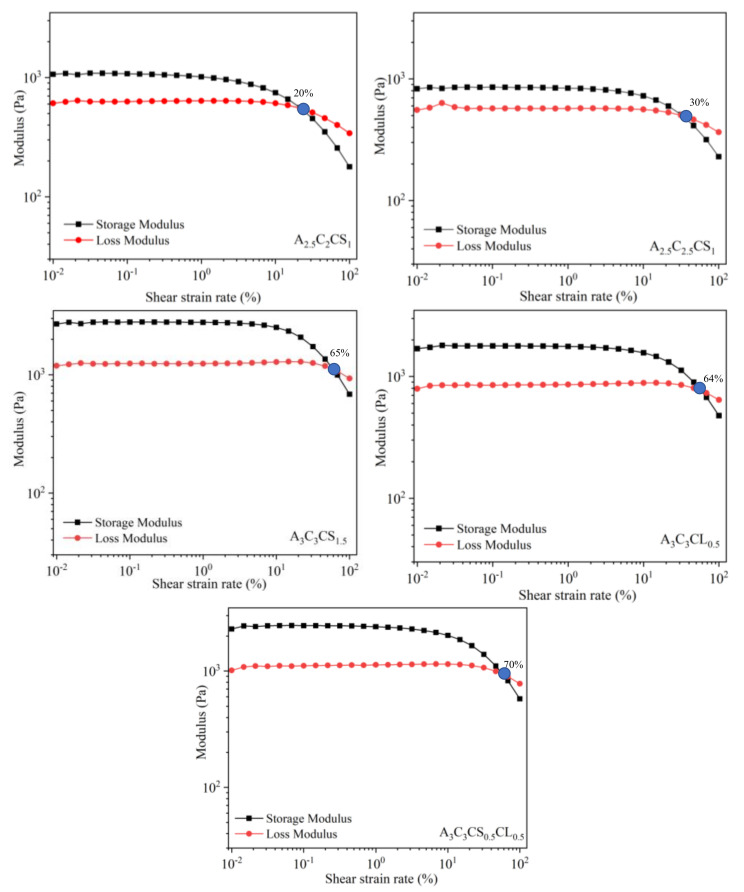
Storage and loss modulus and corresponding gelation point for various compositions. Similar scale is considered ranging from 0.009 to 125% along *X*-axis and 30–3500 Pa along *Y*-axis for all compositions.

The amplitude sweep test is normally performed at a single frequency (1 Hz was used in this study). This test defines the linear region of the material under a subsequent frequency sweep. The deformation amplitude or alternatively the shear stress amplitude is varied while the frequency is kept constant during the test. A complex modulus ($G^{*}=G^{\prime }+iG\prime \prime$) comprised with storage ($G^{\prime },$ solid-like) and loss ($G^{\prime \prime },$ liquid-like) modulus resulted from this test. With less shear strain, solid-like, state-dominated, liquid-like state for all compositions, and domination continued until a certain level before they got intersected, i.e., the gel-point. The linear viscoelastic range (LVR) indicates the range at which the test can be conducted without destroying the structure of the sample, i.e., suspension, preserves the sedimentation without permanent deformation. The strain rate at the LVR and corresponding $G^{\prime }$(yield stress) and $G^{\prime \prime }$values are listed in Table 5. All of them were identified from Figure 6. All 5 compositions showed a ‘gel structure’ because they showed $G^{\prime }>G\prime \prime$ at the LVR region. When shear strain exceeded the intersection point (which is called the flow point), the liquid-like phase started dominating the solid-like phase and caused the material to flow. This flow stress value is helpful to understand the relationship between extrusion pressure and material flow. The effective pressure must suppress this LVR strain rate to extrude the material through the nozzle. In the co-existence of liquid and solid-like phases, increasing the percentage of solid content and cross-linker pushes the LVR boundary, as shown in Figure 6. Therefore, $A_{3}C_{3}{\mathrm{CS}}_{0.5}{\mathrm{CL}}_{0.5}$ reached the gel-point at 70% shear strain where $A_{3}C_{3}{\mathrm{CS}}_{1.5}$, $A_{3}C_{3}{\mathrm{CL}}_{0.5}$, $A_{2.5}C_{2.5}{\mathrm{CS}}_{1}$ and $A_{2.5}C_{2}{\mathrm{CS}}_{1}$ reached at 65%, 64%, 30%, and 20% shear strain, respectively. The continuous drop of $G^{\prime }$after the LVR indicates a gradual breakdown of the internal bonds for all the compositions. 

#### 3.1.3. Three-Point Thixotropic Test: The Recovery Rate

**Figure 7:**
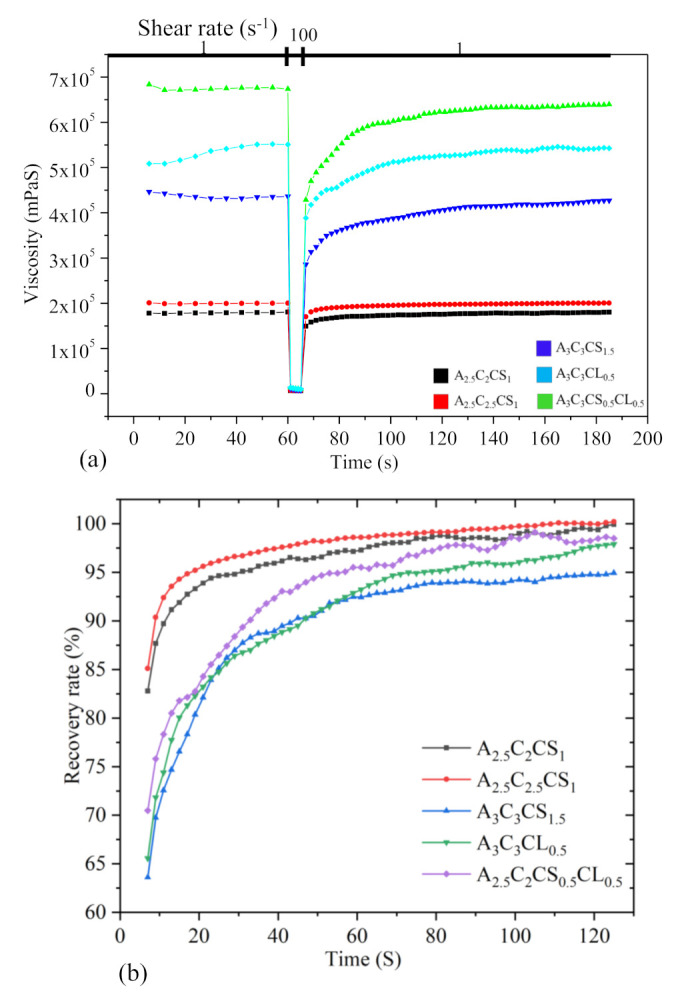
(**a**) Results of three-point thixotropic test and (**b**) Percentage of recovery rate for various compositions. All data points were calculated averaging three sample points.

A three-point-interval thixotropy test was conducted on all the compositions to determine the recovery rate after extruding the hydrogels. This information is critical before the printing begins because it is directly related to the shape fidelity of the filament. In this test, the first interval imitates the at-rest state of the sample, the second interval resembles the hydrogel decomposition under high shear, i.e., hydrogel experiences high shear during extrusion, and the third interval reflects the structure retention after hydrogel extrusion, as shown in Figure 7. 

In the first interval, a shear rate of 1.0 s^−1^ was applied for 60 s. After that, the shear rate was increased to 100 s^−1^ for 5 s. Finally, the shear rate was reduced to 1.0 s^−1^ and held until 120 s. The recovery rates of $A_{3}C_{3}{\mathrm{CS}}_{1.5}$, $A_{3}C_{3}{\mathrm{CL}}_{0.5},{\;A}_{3}C_{3}{\mathrm{CS}}_{0.5}{\mathrm{CL}}_{0.5}$, $A_{2.5}C_{2}{\mathrm{CS}}_{1}$, and $A_{2.5}C_{2.5}{\mathrm{CS}}_{1}$ after 5 s were 64%, 66%, 70%, 83%, and 85%, and they increased to 94%, 98%, 98.5%, 99.5%, and 99.5% after 120 s. A shear rate of 100 s^−1^ was applied to the at-rest hydrogel after 60 s, which broke down the initial network structures of the hydrogel and demonstrated an abrupt reduction of viscosity at 61 s, shown in Figure 7a. After the hydrogel extrusion through the nozzle at a certain shear rate, it took time to recover the internal network. Therefore, when the shear rate was reduced to 1.0 s^−1^ from 100 s^−1^, the amount of viscosity for all the compositions was lower than the initial stage of the tests. In the case of $A_{3}C_{3}{\mathrm{CS}}_{1.5}$, $A_{3}C_{3}{\mathrm{CL}}_{0.5},{\;\mathrm{and}\;A}_{3}C_{3}{\mathrm{CS}}_{0.5}{\mathrm{CL}}_{0.5}$, when shear was released (at time 61 s), a portion of the bond remained unrecovered, but eventually, it recovered over time (at time 120 s). Even $A_{2.5}C_{2}{\mathrm{CS}}_{1}$ and $A_{2.5}C_{2.5}{\mathrm{CS}}_{1}$ were showing very promising recovery rates. There is a high chance they will demonstrate over deposition with the same applied pressure, which will result in poor shape fidelity due to lower initial viscosity when compared to the other considered compositions. Therefore, the application based (either good cell viability or better shape fidelity) material can be selected depending on the initial viscosity and recovery rate.

### 3.2. Scanning Electron Microscope

**Figure 8:**
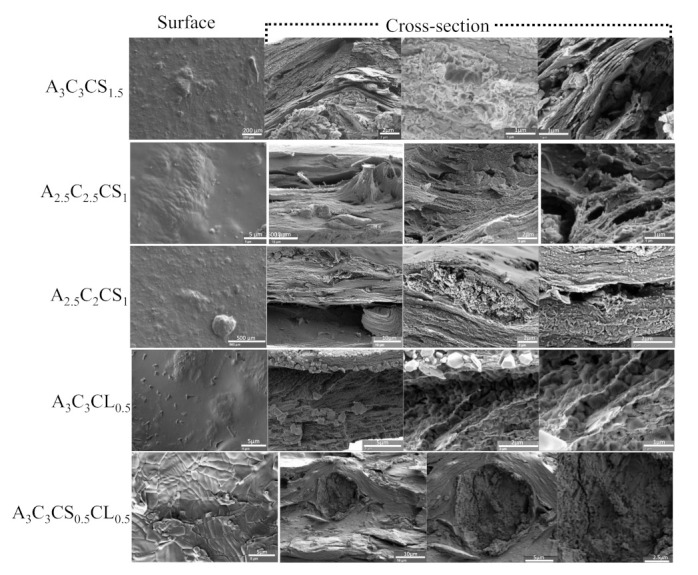
Microstructural investigation on various compositions using a scanning electron microscope.

The surface and cross-sectional morphology of $A_{3}C_{3}{\mathrm{CS}}_{1.5}$, $A_{3}C_{3}{\mathrm{CL}}_{0.5},{\;A}_{3}C_{3}{\mathrm{CS}}_{0.5}{\mathrm{CL}}_{0.5}$, $A_{2.5}C_{2}{\mathrm{CS}}_{1}$, and $A_{2.5}C_{2.5}{\mathrm{CS}}_{1}$ were analyzed by scanning electron microscopy (SEM), as shown in Figure 8. Surface morphologies of all compositions turned out almost smooth, whereas the cross-sections showed crystalline and fiber type morphology. Moreover, cross-sectionally, all the compositions showed porous morphology with various pore sizes. Therefore, there is a high chance that the transmission of required nutrients and exchange of gases of the encapsulated cells in those compositions will be allowed. However, the presence of CaSO_4_ in the compositions creates less cross-linking and eventually results in larger porosity compared to the presence of CaCl_2_. Morphologically, a higher percentage of CMC showed larger micro-porosity, which aligns with our earlier investigation [62].

### 3.3. 3D Printing 

The hybrid hydrogels were extruded by applying air pressure. The materials started flowing depending on the amount of applied pressure. Once the materials overcame the yield stress, they passed through the nozzle. Even flowing materials experience high shear stress. The shear-thinning behavior allows a smooth release with relatively low shear stress on the encapsulated cells.

#### 3.3.1. Bi-Layer Printing and Testing

**Figure 9:**
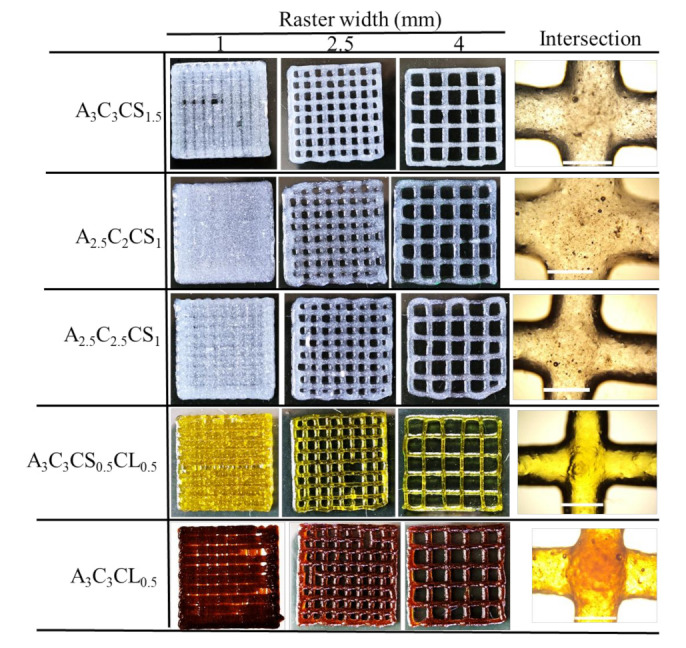
Bi-layer scaffolds having various raster widths and intersection of the filaments, These images were used to determine the printability of all compositions for pore size 2.5 mm × 2.5 mm, and filament widths for all compositions and compared with respect to actual nozzle size.

**Figure 10:**
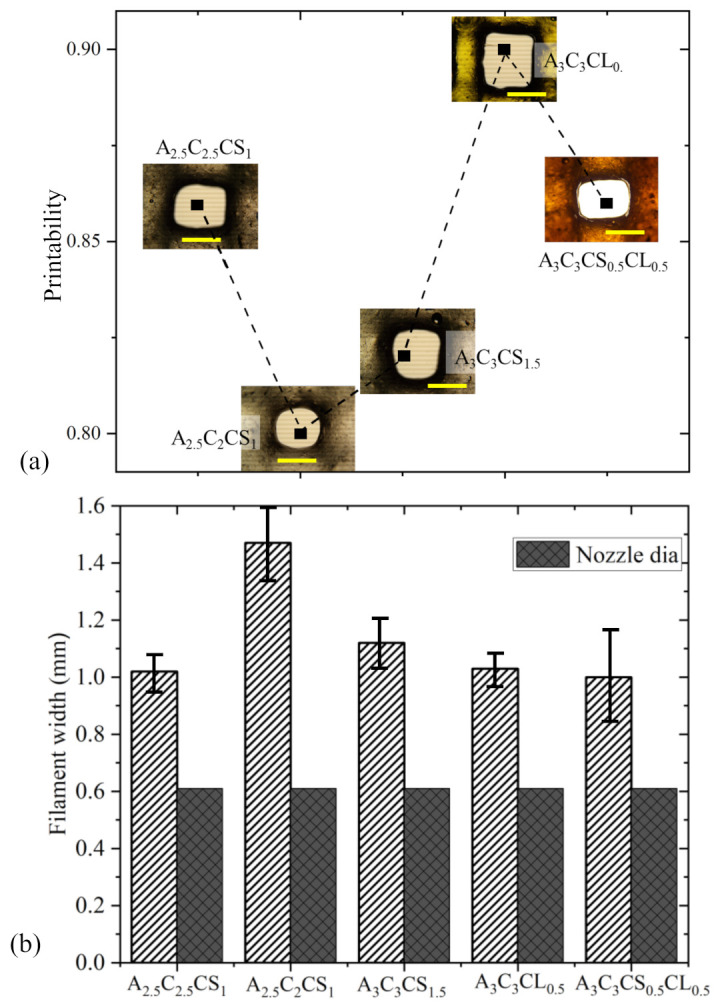
(**a**) Printability of all compositions for a pore size of 2.5 mm × 2.5 mm, and (**b**) Filament widths for all compositions compared to the actual nozzle size. It showed a change ranging from 64–141% with respect to the actual nozzle diameter we extruded through.

Acellular bi-layer scaffolds were fabricated with the compositions of $A_{3}C_{3}{\mathrm{CS}}_{1.5}$, $A_{3}C_{3}{\mathrm{CL}}_{0.5},{\;A}_{3}C_{3}{\mathrm{CS}}_{0.5}{\mathrm{CL}}_{0.5}$, $A_{2.5}C_{2}{\mathrm{CS}}_{1}$, and $A_{2.5}C_{2.5}{\mathrm{CS}}_{1}$ for investigating their manufacturability or printability, as shown in Figure 9 and its result in Figure 10. Those figures indicate that by increasing the percentage of CMC and cross-linker in the composition, the diffusion of the filament decreased. This indicates better geometry holding capacity. Therefore, $A_{2.5}C_{2}{\mathrm{CS}}_{1}$ showed the most deviation (141%) of its filament width where $A_{3}C_{3}{\mathrm{CS}}_{0.5}{\mathrm{CL}}_{0.5}$ showed the least deviation (64%) compared to the nozzle diameter. Therefore, the pores generated by $A_{2.5}C_{2}{\mathrm{CS}}_{1}$ and $A_{3}C_{3}{\mathrm{CS}}_{0.5}{\mathrm{CL}}_{0.5}$ are almost circular and square with a printability value of 0.80 and 0.86, respectively. $A_{3}C_{3}{\mathrm{CS}}_{1.5}$ and $A_{3}C_{3}{\mathrm{CL}}_{0.5}$ showed almost similar filament width deviation (67% and 69%, respectively), which was also supported by their viscosity data. Even numerically, $A_{3}C_{3}{\mathrm{CL}}_{0.5}$ and $A_{3}C_{3}{\mathrm{CS}}_{0.5}{\mathrm{CL}}_{0.5}$ showed better printability and filament width, $A_{3}C_{3}{\mathrm{CS}}_{1.5}$ showed smooth surface roughness and a defined filament intersection morphologically. This indicates the smooth flow of the material. The presence of CaCl_2_ in $A_{3}C_{3}{\mathrm{CL}}_{0.5}$ and $A_{3}C_{3}{\mathrm{CS}}_{0.5}{\mathrm{CL}}_{0.5}$ may create a non-uniform distribution of cross-linking and make the material surface rough during extrusion [63]. 

#### 3.3.2. Collapse Test

**Figure 11:**
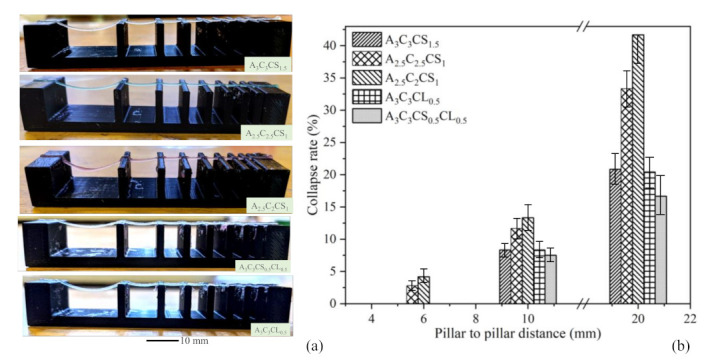
(**a**) Collapse test and (**b**) test result. From 1–5 mm pillar-to-pillar distance, none of the compositions showed collapse. We observed collapse started with a pillar-to-pillar distance of 6.0 mm, and it increased with increasing the pillar-to-pillar distance. At a pillar-to-pillar distance of 20.0 mm, we observed the collapse rate ranging from 20.83 to 41.67%.

The deflection of suspended filaments was measured using a collapse test. The collapse rate was determined by the ratio of deflected (A_a_) and undeflected space (A_t_). Increasing the percentage of solid content and cross-linker lead to a reduction in deflection, as shown in Figure 11a. As a quantitative comparison, the compositions $A_{3}C_{3}{\mathrm{CS}}_{1.5}$, $A_{3}C_{3}{\mathrm{CL}}_{0.5},\;$and $A_{3}C_{3}{\mathrm{CS}}_{0.5}{\mathrm{CL}}_{0.5}$ did not show any collapse up to the 6 mm pillar-to-pillar distance while $A_{2.5}C_{2.5}{\mathrm{CS}}_{1}$ and $A_{2.5}C_{2}{\mathrm{CS}}_{1}$ showed only 2.78% and 4.17% collapse rate, respectively, at 6 mm pillar-to-pillar distance. The collapse rate increased with an increased pillar-to-pillar distance for all compositions. For example, $A_{3}C_{3}{\mathrm{CS}}_{1.5}$, $A_{2.5}C_{2.5}{\mathrm{CS}}_{1}$, and $A_{2.5}C_{2}{\mathrm{CS}}_{1}$showed 20.83%, 33.33%, and 41.67% collapse rates, respectively, at the 20 mm pillar-to-pillar distance. $A_{3}C_{3}{\mathrm{CS}}_{1.5}$ and $A_{3}C_{3}{\mathrm{CL}}_{0.5}\;$showed almost similar collapse rates for all pillar-to-pillar distances. It is quite clear from the result, as shown in Figure 11b, that we can use all of the compositions to make large structures. 

#### 3.3.3. Mechanical/Compressive Test

**Figure 12:**
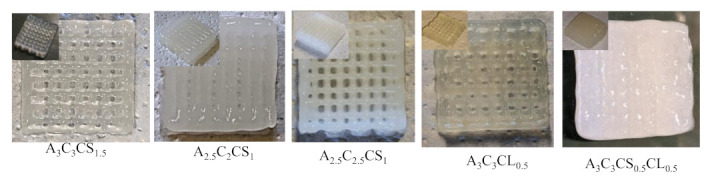
Samples used to conduct compressive tests. We printed three samples with each composition and observed very minimal differences printed with the same composition. Mechanical test data represents the average of three sample data.

**Figure 13:**
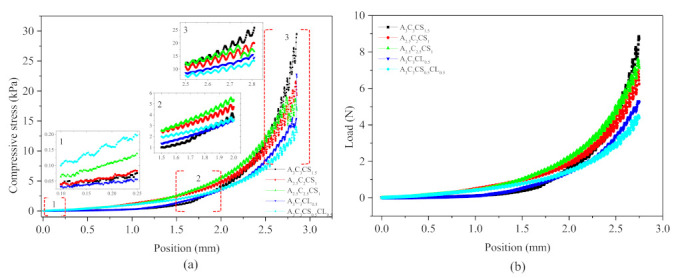
(**a**) Compressive stress and (**b**) Corresponding load with respect to the position change of the load cell. Each data point was calculated by averaging three sample points.

The mechanics of the bio-printed cubes (10 layers) with a size of 20 mm × 20 mm were analyzed for their structural fidelity by comparing the compressive modulus of all compositions, as shown in Figure 12 and Figure 13. Results showed that at the beginning of compression, $A_{3}C_{3}{\mathrm{CS}}_{0.5}{\mathrm{CL}}_{0.5}$ showed more compressive strength than other compositions. Interestingly, $A_{3}C_{3}{\mathrm{CS}}_{1.5}$ showed 25%, 40%, 55%, and 65% better compressive stress at the end with respect to $A_{2.5}C_{2}{\mathrm{CS}}_{1}$, $A_{2.5}C_{2.5}{\mathrm{CS}}_{1}$, $A_{3}C_{3}{\mathrm{CL}}_{0.5}$, and $A_{3}C_{3}{\mathrm{CS}}_{0.5}{\mathrm{CL}}_{0.5}$. This indicates that it has a higher chance of maintaining better shape fidelity. Figure 12 shows that all constructs, except one bio-printed by $A_{3}C_{3}{\mathrm{CS}}_{0.5}{\mathrm{CL}}_{0.5},$ were stable enough to overcome the gravity and will allow for post-crosslinking to permanently stabilize the structures. 

#### 3.3.4. Large Scale Scaffold Fabrication

**Figure 14:**
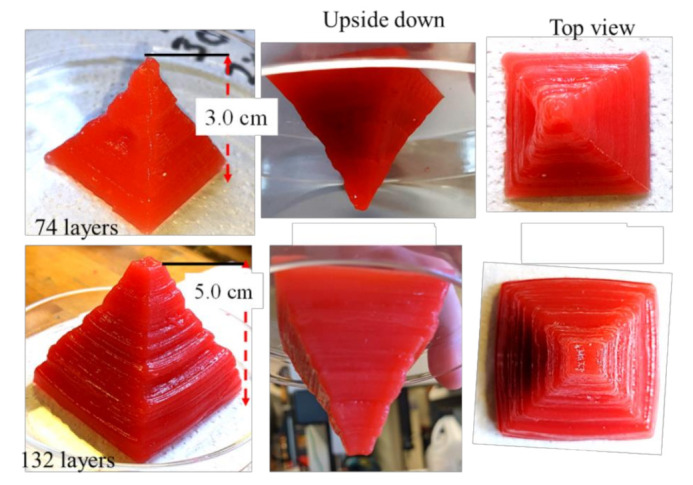
Pyramid structure fabricated using $A_{3}C_{3}{\mathrm{CS}}_{1.5}$. It preserved the external and internal geometric fidelity and showed minimal discrepancy with respect to the digital model we used for fabrication.

**Figure 15:**
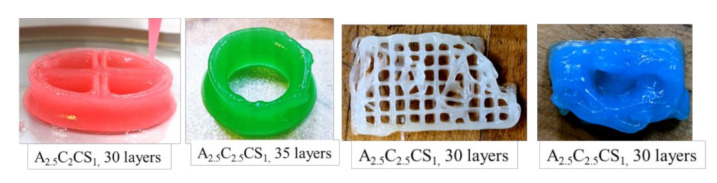
Scaffolds printed with the compositions of A_2.5_C_2.5_CS_1_ and A_2.5_C_2_CS_1_. All scaffolds preserved the external and internal geometric fidelity and showed minimal discrepancy with respect to the digital model we used for fabrication.

Characterization tests such as rheological tests, filament width and fusion tests, collapse tests, and microstructural tests demonstrated that all pre-crosslinked compositions have the potential to fabricate large scaffolds. Compositions pre-crosslinked with CaSO_4_ were used to fabricate acellular scaffolds to demonstrate the 3D printability of large and freeform scaffolds. Two pyramidal scaffolds were fabricated with a base dimension of 30 mm × 30 mm and 50 mm × 50 mm and a build height of 30 mm (74 layers) and 50 mm (132 layers), respectively, using the composition $A_{3}C_{3}{\mathrm{CS}}_{1.5}$, as shown in Figure 14. We also used compositions $A_{2.5}C_{2.5}{\mathrm{CS}}_{1}$ and $A_{2.5}C_{2}{\mathrm{CS}}_{1}$ to fabricate various scaffolds, as shown in Figure 15. It is clear from those figures that compositions exhibit the capability to maintain good inter-layer diffusion and the shape fidelity of the fabricated scaffold. 

### 3.4. Swelling Test

**Figure 16:**
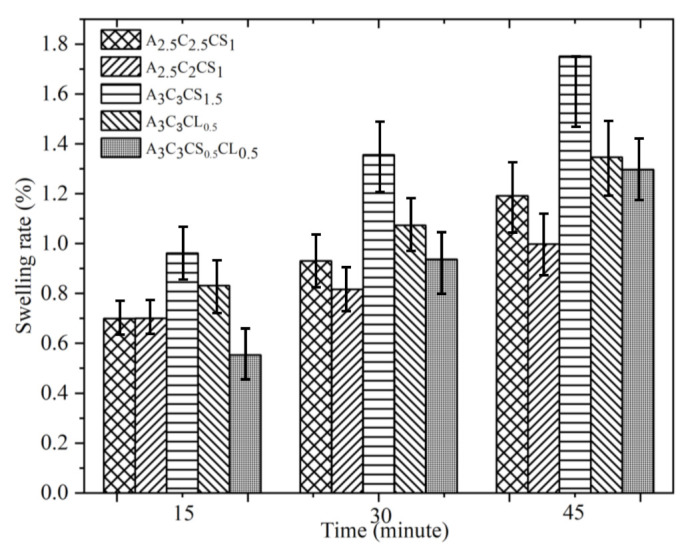
Result of swelling test for various compositions at different times. Statistically, it showed a significate difference (*p* < 0.05) with respect to the various times we used (15, 30, 45 min).

The swelling rate defines the degree of water uptake of the bio-ink, which indicates the applicability of the bio-ink in biological applications. The higher percentage of swelling specifies greater retention of nutrients during the incubation period, which better mimics the native tissue. The swelling behavior and mesh size also play an important role in regulating nutrient transport, cell–cell and cell–substrate interactions, cell viability, and proliferation [63,64]. Additionally, the exchange of required gases and the removal of waste is also governed by the swelling rate [65]. However, a higher swelling ratio in quick time will expedite the pore closure [66] and eventually impair the geometric fidelity of the scaffold in the incubation period [67]. In this experiment, all compositions showed an increasing trend of the swelling rate with time, as shown in Figure 16. There was no significant difference in swelling rate among various compositions at any specific time. This result indicates that all compositions can preserve the pore geometry and consequently the shape fidelity of the incubated scaffolds for a longer time. They can produce better internal and external architecture of the matured scaffold towards the end of the incubation period.

### 3.5. Bacterial Growth

The Most Probable Numbers assay is a viability-based assay. E. coli can only be quantified if they grow, as growth is the endpoint for the assay. The data presented here represent a statistical estimate of the number of viable E. coli present in the hydrogel at the times sampled. The Most Probable Number of *Escherichia coli* in scaffold $A_{3}C_{3}{\mathrm{CS}}_{0.5}{\mathrm{CL}}_{0.5}$ at time zero and 24 h were 3.8 × 10^6^ (±1.1 × 10^6^) and 1.1 × 10^6^ (±1.3 × 10^5^), respectively, where standard errors at time zero and 24 h were 1.1 × 10^6^ and 1.3 × 10^5^, respectively. The *p*-value associated with the paired T-test conducted was 0.29, which exceeds 0.05, failing to disprove the statistical hypothesis that the means were the same. We conclude that scaffold $A_{3}C_{3}{\mathrm{CS}}_{0.5}{\mathrm{CL}}_{0.5}$ does not support the growth of *E. coli* when provided with solely the scaffold, water, and the optimal temperature for E. coli (37 °C). These results may suggest that this scaffold is intrinsically antimicrobial [68]. 

## 4. Discussion

A set of hybrid hydrogels were pre-crosslinked, applying CaCl_2_ and CaSO with various percentages (0.5–1.5%). Rheological test results showed viscosity and gelation point were percentage of solid content and cross-linker dependent. Therefore, $A_{3}C_{3}{\mathrm{CS}}_{0.5}{\mathrm{CL}}_{0.5}$ showed the highest viscosity, and we achieved the gelation point at a 70% shear rate. All pre-crosslinked hydrogels showed shear thinning behavior (*n* < 1). Three-point thixotropic tests demonstrated that all the compositions could retain more than 90% of their initial viscosity after 120 sec. However, $A_{2.5}C_{2}{\mathrm{CS}}_{1}$ and $A_{2.5}C_{2.5}{\mathrm{CS}}_{1}$ showed a relatively better recovery rate right after the extrusion due to their less solid content and cross-linker percentage, which eventually created fewer inter-molecular bonds compared to the other three compositions. However, the same applied pressure may push toward over deposition of $A_{2.5}C_{2}{\mathrm{CS}}_{1}$ and $A_{2.5}C_{2.5}{\mathrm{CS}}_{1}$ and eventually, will result in poor shape fidelity due to lower initial viscosity than other considered compositions. Therefore, the choice of the compositions can be application-driven, such as either good cell viability or better shape fidelity. The microstructural analysis represented the presence of porous morphology with various pore sizes. Pore morphology and size were cross-linker and CMC dependent. Therefore, $A_{3}C_{3}{\mathrm{CS}}_{1.5}$ and $A_{2.5}C_{2.5}{\mathrm{CS}}_{1}$ showed better micro-porosity, which indicates more potential to allow the transmission of required nutrients and exchange of gases of the encapsulated cell into those compositions. From the bi-layer printing and collapse test, it is clear that $A_{2.5}C_{2}{\mathrm{CS}}_{1}$ has 141% deviation of the filament width, where $A_{3}C_{3}{\mathrm{CS}}_{1.5}$, $A_{3}C_{3}{\mathrm{CL}}_{0.5}$, and $A_{3}C_{3}{\mathrm{CS}}_{0.5}{\mathrm{CL}}_{0.5}$ showed almost similar filament deviation. However, $A_{3}C_{3}{\mathrm{CS}}_{1.5}$ showed smooth surface texture and a defined filament intersection morphologically. CaCl_2_ is readily soluble in water, which often leads to an uncontrolled release of Ca^2+^ ions and therefore results in a heterogeneous cross-linking. The extra amount of Ca^2+^ can create a random cross-linking that leads to the use of higher extrusion pressure while printing. Therefore, even $A_{3}C_{3}{\mathrm{CS}}_{0.5}{\mathrm{CL}}_{0.5}$ showed good viscosity, its bi-layer intersection showed a rough texture indicating the intermittent flow through the nozzle. Since CaSO_4_ has a higher Young’s modulus and equilibrium modulus, it has better mechanical properties. Compositions cross-linked with CaSO_4_ exhibit better structural integrity after printing than those cross-linked with CaCl_2_ [41,42]. The compressive test result with 10 layered scaffolds was interesting where at the beginning, $A_{3}C_{3}{\mathrm{CS}}_{0.5}{\mathrm{CL}}_{0.5}$ showed more compressive strength than other compositions. However, $A_{3}C_{3}{\mathrm{CS}}_{1.5}$ showed 25%, 40%, 55%, and 65% better compressive strength at the end with respect to $A_{2.5}C_{2}{\mathrm{CS}}_{1}$, $A_{2.5}C_{2.5}{\mathrm{CS}}_{1}$, $A_{3}C_{3}{\mathrm{CL}}_{0.5}$, and $A_{3}C_{3}{\mathrm{CS}}_{0.5}{\mathrm{CL}}_{0.5}$ indicates it has more chance to carry self-weight, which can eventually maintain better shape fidelity. $A_{3}C_{3}{\mathrm{CS}}_{0.5}{\mathrm{CL}}_{0.5}$ failed to maintain the structural integrity and overcome the gravity due to intermittent flow (under and over-deposition). All three compositions pre-crosslinked with CaS0_4,_ such as $A_{3}C_{3}{\mathrm{CS}}_{1.5}$, $A_{2.5}C_{2.5}{\mathrm{CS}}_{1}$ and $A_{2.5}C_{2}{\mathrm{CS}}_{1}$ were used to fabricate large scale scaffolds. We successfully printed a pyramidal scaffold with 50 mm (132 layers) build height using $A_{3}C_{3}{\mathrm{CS}}_{1.5}$ which ensured the geometric fidelity of the scaffold. Finally, a bacterial growth test with the MNP method indicates that $A_{3}C_{3}{\mathrm{CS}}_{0.5}{\mathrm{CL}}_{0.5}$ can be used as a potential bio-ink prohibiting harmful bacterial growth during and after the bioprinting process. 

Table 6 shows a comparison between proposed and various existing hybrid hydrogels in terms of solid content, percentage of pre-crosslinker and subsequent viscosity, applied pressure, print speed, and the number of layers printed. It is clear from this comparison that we can achieve better viscosity with the application of viscosity enhancer and pre-crosslinker simultaneously, ensuring less solid content. Therefore, $A_{3}C_{3}{\mathrm{CS}}_{1.5}$ showed almost double viscosity with less solid content (6%) and 1.5% CaSO_4_ (which is a weaker cross-linker than CaCl_2_) compared to [4]. Moreover, an almost similar amount of viscosity as resulted in [4] can be achieved with only 5% solid content and 1% CaSO_4_. Even the filament morphology was reported, the shape fidelity of 3D structure printed with 3% solid content and 0.28% CaCl_2_ pre-crosslinker was not reported in [69], which did not allow us the opportunity to compare with proposed compositions. We started extruding our compositions with higher pressure and reduced them gradually throughout the printing process. Moreover, we printed comparatively faster (50 mm/s) than other hybrid hydrogels listed in Table 6, which may affect the applied pressure minimally on the extrusion.

## 5. Conclusions

In this research, we hypothesized that applying a pre-crosslinker to a hybrid hydrogel having a smaller amount of solid content can result in equivalent or better mechanical and rheological properties and, eventually, better printability and geometric fidelity. Hybrid hydrogels (alginate and CMC) pre-crosslinked with CaCl_2_ and CaSO_4_ showed better viscosity, compressive strength, and, consequently, improved printability and shape fidelity. Advanced analytical analysis by amplitude test, three-point thixotropic test, and microstructural test indicated the important properties such as gelation point, recovery rate, and presence of micro-porosity of various compositions. However, CaSO_4_ as a pre-crosslinker showed better surface finish, interlayer diffusion, micro-porosity, and better compressive strength. The future direction of this research is finding out the effect of bacteria on $A_{3}C_{3}{\mathrm{CS}}_{1.5},$  $A_{2.5}C_{2}{\mathrm{CS}}_{1},$ and $A_{2.5}C_{2.5}{\mathrm{CS}}_{1}$. To our best knowledge, for the first time, we proposed hybrid hydrogels consisting of alginate and carboxymethyl cellulose (CMC) (high and low viscosity) for the extrusion-based bioprinting process [48,49,50,51]. We reported a range of cell viability from 80 to 91% in those various compositions with different printing conditions. Based on those results and extreme printing conditions used in [21,70], we are expecting to have viable cells into our proposed pre-crosslinked bio-ink at different printing conditions. In future, a set of human living cells (especially eukaryotic functional cells) will be encapsulated with all $A_{3}C_{3}{\mathrm{CS}}_{1.5}$, $A_{2.5}C_{2.5}{\mathrm{CS}}_{1}$ and $A_{2.5}C_{2}{\mathrm{CS}}_{1}$compositions, and the cell viability and proliferation will be determined. We believe this pre-crosslinking-based physical and mechanical properties controlling technique can open a new avenue for 3D bio-fabrication of scaffolds, ensuring proper geometry. 

## Figures and Tables

**Table 1:** Formulated hybrid hydrogels with weight percentages of various components.

% (*w*/*v*) Alginate, A			% (*w*/*v*) of CMC, C	% (*w*/*v*) CaSO_4,_ CS	% (*w*/*v*) CaCl_2,_ CL	Formula
LV	MV	Total				
1	1.5	2.5	2.5	1	0	A_2.5_C_2.5_CS_1_
1	1.5	2.5	2	1	0	A_2.5_C_2_CS_1_
1	2	3	3	1.5	0	A_3_C_3_CS_1.5_
1	2	3	3	0	0.5	A_3_C_3_CL_0.5_
1	2	3	3	0.5	0.5	A_3_C_3_CS_0.5_CL_0.5_

**Table 2:** An overview of all rheological tests.

Rheological Tests	Process Variables	Recorded Outcome
Steady rate sweep	Shear rate(s^−1^): 0.1 to 500	Flow curve, viscosity, shear stress, shear-thinning behavior
Amplitude sweep	Shear strain (%): 0.1 to 100	Storage modulus (G’) and loss modulus (G”), loss tangent (tanδ)
3iTT	Time(s)/Shear rate (s^−1^): 0–60/1, 61–65/100, 66–185/1	Recovery rate of the hydrogel

**Table 3:** Process parameters used in this paper.

Process Parameters	Value/Characteristics
Nozzle diameter	610 µm
Layer height	250 µm
Infill pattern	Zig-zag and contour-parallel
Infill density	50%
Print speed	50 mm/s
Air pressure	15–20 psi
Print distance	0.405 mm

**Table 4:** Shear-thinning co-efficient (*n* and k) and shear rates during extrusion and corresponding shear stresses and viscosities.

Compositions	Filament Width (µm)	Average Velocity (mm/s)	*n*	K (mPa.S^n^)	Shear Rate (s^−1^)	Shear Stress (mPa)	Viscosity (mPa.S)
A_2.5_C_2_CS_1_	1470	120.5	0.23	212,330	2867.53	1,376,913	480.17
A_2.5_C_2.5_CS_1_	1120	91.8	0.24	249,760	2175.76	1,537,522	706.66
A_3_C_3_CS_1.5_	1020	83.6	0.03	608,257	10,012.77	800,590.4	79.96
A_3_C_3_CL_0.5_	1030	84.4	0.07	603,397	4630.61	1,115,859	240.97
A_3_C_3_CS_0.5_CL_0.5_	1000	81.9	0.02	775,660	14,540.86	935,665.6	64.35

**Table 5:** Yield and flow point and corresponding yield and flow stress.

	A_2.5_C_2_CS_1_	A_2.5_C_2.5_CS_1_	A_3_C_3_CS_1.5_	A_3_C_3_CL_0.5_	A_3_C_3_CS_0.5_CL_0.5_
% strain at yield point	0.068	0.465	0.681	0.216	0.317
Yield stress (mPa)	1083	848	2789	1785	2453
G” in yield point (mPa)	637	572	1243	853	1120
% strain at flow point	20	30	65	64	70
Flow stress (mPa)	559	509	1100	1006	960

**Table 6:** Comparison between proposed and various existing hybrid hydrogels in terms of solid content, pre-crosslinker percentage and subsequent viscosity, applied pressure, and the number of layers printed.

Solid Content (%)	Components	% (*w*/*v*) of Pre-Crosslinker		Viscosity at 1s^−1^ (Pa.S)	Applied Pressure (psi)	Print Speed (mm/s)	No. of Layers Printed	Ref.
		CaCl_2_	CaSO_4_					
12 (3/9)	Alg/MC	0	0	800	58	NA	50	[21]
8	MC	0	0	500	35	10	42	[19]
12(3/3/6)	Lap/Alg/MC	0	0	1000	13.78	8–10	30	[70]
8	Alg	1	0	300	29	10	NA	[4]
3	Alg	0.28	0	125	NA	0.75	NA	[69]
8 (4/4)	Alg/CMC	0	0	500	8	4	40	[62]
6(3/3)	Alg/CMC	0.5	0.5	776	20	50	10	Proposed
6(3/3)	Alg/CMC	0.5	0	603	18	50	10	
6(3/3)	Alg/CMC	0	1.5	608	18	50	132	
5(2.5/2.5)	Alg/CMC	0	1	250	16	50	35	
4.5(2.5/2)	Alg/CMC	0	1	212	15	50	30	

## Data Availability

Not applicable.
